# Creativity as a Means to Well-Being in Times of COVID-19 Pandemic: Results of a Cross-Cultural Study

**DOI:** 10.3389/fpsyg.2021.601389

**Published:** 2021-03-09

**Authors:** Min Tang, Sebastian Hofreiter, Roni Reiter-Palmon, Xinwen Bai, Vignesh Murugavel

**Affiliations:** ^1^Institute for Creativity and Innovation, University of Applied Management, Ismaning, Germany; ^2^Department of Psychology, University of Nebraska Omaha, Omaha, NE, United States; ^3^CAS Key Laboratory of Behavioral Science, Institute of Psychology, Chinese Academy of Sciences, Beijing, China

**Keywords:** COVID-19, creative process engagement, creative growth, social connectedness, employees, social well-being, flourishing well-being, cross-cultural study

## Abstract

The coronavirus disease 2019 (COVID-19) pandemic has brought about unprecedented uncertainty and challenges to the worldwide economy and people’s everyday life. Anecdotal and scientific evidence has documented the existence of a positive relationship between the experience of crisis and creativity. Though this appears to be ubiquitous, the crisis-creativity-well-being relationship has not been sufficiently examined across countries and using a working adult sample. The current study drew on a sample consisting of 1,420 employees from China (*n* = 489, 40% females), Germany (*n* = 599, 47% females), and the United States (*n* = 332, 43% females) to examine whether creativity can function as an effective means to cope with crisis and to achieve both flourishing and social well-being. Multivariate analyses showed that perceived impact of COVID-19 was positively related to creative process engagement, which was positively related to employees’ self-reported creative growth. Creative growth was associated with a higher level of flourishing well-being. This sequential mediation model was significant across the three samples. Creativity also mediated the relationship between perceived impact of COVID-19 and social well-being (social connectedness), but this connection was only found for the Chinese sample. Further data analyses revealed that individualism moderated this serial mediation model in that the positive coping effect of creativity on both flourishing and social well-being was stronger for individuals who hold more collectivistic views. Results of the study have implications for crisis management, personal development, and positive functioning of individuals and society.

## Introduction

Since the beginning of 2020, the whole world was confronted with the grand challenges posed by the coronavirus disease 2019 (COVID-19) pandemic. The absolutely new, unknown, and uncertain situations that the pandemic has brought about required individuals and organizations to “find new ways to connect creativity, innovation, ethics, and sustainability” if they want to survive this crisis and become stronger and more resilient (Hölzle et al., 2020, p. 195). Though anecdotal and scientific evidence has documented the possible relationship between the experience of crisis/disasters and creativity (e.g., Damian and Simonton, 2014; Orkibi and Ram-Vlasov, 2019), and creativity and well-being (Smith, 2016; Conner et al., 2018), this crisis-creativity-well-being relationship has not been sufficiently examined across countries. The present study aims to answer the following questions: is creativity a means to well-being and social connectedness (SC) when facing a crisis? And is this mediating effect of creativity between crisis and well-being and crisis and SC universal across three different cultures: China, Germany, and the United States?

Well-being is a broad concept, which is composed of three major components: life satisfaction, positive experiences, and negative experiences (Diener, 1984). The focus of the current study is the positive experiences in face of the threat of the COVID-19 pandemic. We chose this focus because studies have shown that people who experience positive feelings most of the time enjoy better health and live longer (Lyubomirsky et al., 2005; Diener and Chan, 2011); they also have better relationships and work more productively (Harter et al., 2010). On the contrary, negative emotions (Huppert, 2009) and relative lack of social relations (Tay et al., 2013) strongly predict overall mortality and disease outcomes. A new study involving 53,524 respondents from 26 countries provides evidence that due to the COVID-19 pandemic, single persons who lack in SC exhibit higher levels of stress than married or cohabiting people (Kowal et al., 2020). Given the specific effect of COVID-19 on SC though the need for distancing and isolation, this study also focuses on SC and attempts to examine the possible mediating effect of creativity in helping people cope with stress and achieve well-being in times of crisis. Following mainstream psychological studies, we define creativity as a human capacity to produce products, ideas, or solutions that are both novel and appropriate (Amabile, 1996; Zhou and Shalley, 2003; Mumford, 2012; Runco and Jaeger, 2012). In the present study, we specifically focus on *functional creativity*, that is, creativity in the service of solving everyday problems (see Cropley and Cropley, 2010) instead of arts-related expressive creativity. We rely on the transformative coping model (TCM; Corry et al., 2014, 2015) as the overarching theoretical framework to explore why creativity can serve as the mechanism through which individuals cope with and thrive from the crisis like the COVID-19 pandemic. Consistent with the transactional model of stress and coping (Lazarus and Folkman, 1984), the TCM distinguishes between the primary and secondary appraisal processes as individuals evaluate the stressful situation. Different from Lazarus and Folkman (1984), however, Corry and colleagues argue that individuals can initiate a process of transformative coping by mobilizing their own inherent human capacities of creativity to cope with adversities. By engaging in creative activities, they harness and amplify their positive feelings about themselves (e.g., perceived personal growth), which will subsequently improve resilience and well-being.

## Theory and Hypotheses

### Crisis, Diversifying Experiences, and Creativity

Crisis is defined as “a disruption that physically affects a system as a whole and threatens its basic assumptions, its subjective sense of self, its existential core” (Pauchant and Mitroff, 1992, p. 15). The disruption that a crisis brings about draws individuals or organizations from their familiar and normal situations. Relatedly, crisis situations can result in “diversifying experiences,” which are defined as “highly unusual and unexpected events or situations that push individuals outside the realm of ‘normality’” (Ritter et al., 2012, p. 961). Diversifying experiences can be positive (e.g., multicultural education, work, or life experience) or negative (e.g., childhood traumatic experience like early parental loss, financial difficulty, social exclusion, or mental illness). Damian and Simonton (2015) propose a model to account for the reason why experiencing adversities are related to creative accomplishments. According to this model, the common function of diversifying experiences, regardless of form, lies in that they push individuals outside the frameworks of their ordinary daily lives, promote cognitive flexibility, and force individuals to embrace new and uncommon ideas (Damian and Simonton, 2014, 2015). After being exposed to highly novel or traumatic events, individuals will find it necessary to reappraise their core beliefs about the self and the world. As a result, they are more willing to make changes in many aspects, such as increased appreciation of life, a fresh look at interpersonal relationships, recognition of personal strength, exploration of new possibilities, or spiritual development, all of which can contribute to the manifestation of creativity (Forgeard, 2013). Indeed, empirical evidence consistently indicates that diversifying experiences, such as mental illness (Ludwig, 1992; Damian and Simonton, 2015), social rejection or social isolation (Akinola and Mendes, 2008; Kim et al., 2013), early parental death or other traumatic experiences during childhood (Simonton, 1994; Damian and Simonton, 2015), war (Orkibi and Ram-Vlasov, 2019), and setbacks in adulthood (Niu and Kaufman, 2005), are associated with a higher level of creativity (for a review, see Damian and Simonton, 2014). Moreover, experiencing adversity may promote motivation to engage in creative endeavors because individuals rely on creative engagement to overcome the constrains and disadvantages caused by adverse events (Cheng et al., 2015; Acar et al., 2019). We therefore propose the following hypothesis:

*H1*: The perceived impact of COVID-19 (PIC) crisis is positively related to creative process engagement (CPE).

### Flourishing and Social Well-Being

Well-being is one of the most enduring topics in psychological investigation. Diener and Seligman (2004, p. 1) defined well-being as “peoples’ positive evaluations of their lives, includes positive emotion, engagement, satisfaction, and meaning.” Ryan and Deci (2001) defined well-being as optimal psychological functioning and experience, and they stressed the differentiation of hedonic approach (focusing on happiness) and eudaimonic approach (focusing on meaning and self-actualization). One typical eudaimonic well-being is flourishing, which is characterized by optimal functioning accompanied by feelings of meaning, engagement, and purpose in life (Ryan and Deci, 2001).

Recently Feeney and Collins (2015) argued that well-being has a social component, which is characterized as an individual’s “… deep and meaningful human connections, positive interpersonal expectations…. (p. 115).” Similarly, Ryan and Deci (2000) maintain that relatedness, the need to feel belongingness and connectedness with others, is an important innate psychological need. Given that lockdown and social distancing have been widely adopted to fight the COVID-19 pandemic, it is of great importance to explore how the COVID-19 crisis affects individuals’ social well-being. In the present study, we follow Feeney and Collins (2015) to include SC as a specific type of social well-being in parallel to the flourishing well-being (FWB).

Lee and Robbins (1998) describe SC as an individual’s sense of belonging and the subjective perception of having close and distant relationships in the social context (e.g., friends, family, strangers, community, or society). SC is associated with diverse psychological outcomes such as increased self-esteem, social identification, cooperative behavior, trust, well-being, life-satisfaction, positive emotions, and decreased depression and anxiety (Lee and Robbins, 1998; Glaeser et al., 2000; Ong and Allaire, 2005; Williams and Galliher, 2006; Lee et al., 2008; Mauss et al., 2011). Individuals who lose their perceived connection to other humans tend to struggle with social roles and responsibilities, giving people the feeling of disconnection, which can lead into stronger isolation (Lee and Robbins, 1995).

### Creativity as the Resource of Enhancing Well-Being

Much of the focus of the work on creativity and well-being stems from work with vulnerable individuals such as those with disabilities, metal-health issues, or aging populations (Cohen et al., 2006; Gostoli et al., 2017; Cera et al., 2018). In fact, the entire field of art therapy has emerged as a result of the perceived connection between creativity and well-being as a way to improve mental-health (Smith, 2016). Smith suggested that art therapy facilitates improvement in mental-health, as it allows patients to experience and verbalize the difficult emotions, provides a distraction, and can lead to positive emotions through the creation process. These mechanisms have also been suggested to apply outside of these vulnerable populations. In recent years, the relationship between creative activities and subjective well-being has also been explored in normal adult population such as medical professionals (Phillips and Becker, 2019) and undergraduate students (Drake, 2019).

Of course, creative engagement is not only limited to artistic activities. Rather, creativity exists across domains (Kaufman and Baer, 2005). Extending the scope of creativity to various everyday creative activities and using experience sampling on a large sample (*n* = 658) of young adults, Conner et al. (2018) found that the engagement in creative activities led to increases in positive affect and flourishing in the day after, supporting the notion that creative engagement leads to increased well-being. Creative engagement, no matter in what form, is usually self-driven and intrinsically motivated, and is one of the key psychological factors that can lead to greater flourishing (Ryan and Deci, 2000). As a result, several reviews have proposed creative activities as an intervention to foster well-being and flourishing (Forgeard and Eichner, 2014; Lomas, 2016). Though the effect of artistic creativity and everyday creativity on well-being has been studied and established, there seems to be no investigation of the effect of problem-solving-focused functional creativity and, specifically, engagement in creative problem-solving processes (Cropley and Cropley, 2010) on well-being. This study attempts to fill this gap by focusing on creative process engagement of employee samples.

Humanistic psychology views creativity as a way to reach wholeness and self-actualization. This type of primary self-actualizing creativity, in the words of Maslow (1962), is a “heritage of every human being (p. 95).” In a similar vein, Rogers (1961) maintained that creativity as an underlying motivational force for growth. The TCM (Corry et al., 2014, 2015) was developed based on the humanistic notion of creativity as well as the transactional model of stress and coping (Lazarus and Folkman, 1984). This model posits that in stressful circumstances, individuals will go through the primary and secondary appraisal processes (Lazarus and Folkman, 1984) to evaluate the stressful situation and resources they could use for coping. Creativity, which can be viewed as such a resource, enables individuals to transform their perspectives on life, provide meaning to a novel situation, and find solutions to problems (Kaufman, 2018).

In the revised TCM, Corry et al. (2015) proposed a *sequential* coping mechanism in which individuals first appraise the stressful event of situation and then apply various coping strategies (e.g., creativity and creative problem solving). The engagement in creative activities will then harness and amplify their positive feelings about themselves (e.g., perceived personal growth), which will subsequently improve resilience and well-being. Following this model, we consider two creativity measures in the current study: creative process engagement and creative growth. Whereas creative process engagement is concerned with participants’ actual involvement in creativity-related processes (i.e., problem identification, information searching, idea generation, and problem solving; Zhang and Bartol, 2010), creative growth is defined as an individual’s perceived increase/growth in creativity or motivation for creativity (Forgeard, 2013). With these two measures, we hope to be able to examine the nuanced contribution of different aspects of creativity in the relationship between crisis and well-being. Based on the existing literature, we propose the following:

*H2*: Creative process engagement (CPE) is positively related to perceived creative growth (PCG).

We also expect that creativity will yield positive impact on social well-being. Because of the existence of sporadic, in extreme cases of “lone geniuses” such as van Gogh, Tesla, and Beethoven, the link between loneliness and creativity has become almost a cliché. However, this “lone genius” myth, though seemingly ubiquitous, has been debunked (Glăveanu, 2020). Actually, it has not been scientifically examined until recently. Research evaluating team creativity has long suggested that individuals working well in teams and forming close and positive relationships result in improved creativity (Reiter-Palmon and Paulus, 2020). In addition, recent work on creativity and social relationship has found that both creators and students were more creative with better social relationships such as romantic relationships or friendships (McKay et al., 2017; Lebuda and Csikszentmihalyi, 2020). Creativity as a precursor of SC can mainly be found in therapeutic contexts. Using creativity-based therapeutic activities such as visiting an art museums (e.g., Bennington et al., 2016) or scrapbooking in groups (e.g., Fiorito et al., 2020) is shown to promote SC. Sharing and talking about creative experiences and creative products seem to improve SC. This connecting effect seems to be particularly important in crisis times such as the COVID-19 pandemic when quarantine and social distancing have become some of the most common epidemic prevention and control measures across countries and this can lead to social isolation and feelings of loneliness (Killgore et al., 2020). Based on the above arguments, we propose the following hypotheses:

*H3a*: PCG is positively associated with FWB.
*H3b*: PCG is positively associated with SC.

### Individualism vs. Collectivism, Well-Being, and Social Connectedness

Individualism puts individuals in the center of attention and emphasizes personal interests, individual values/goals, and independence of individuals. Collectivism, in contrast, prioritizes the group over the self and underlines collective interests, common values/goals, and interdependence of individuals (Hofstede, 1980; Triandis, 1995). Individualism and collectivism are among the most frequently studied cultural dimensions in psychological and social sciences (see a review, Oyserman et al., 2002; Taras et al., 2010) and have been often applied to explain the differences between the East and the West in creativity studies as well (e.g., Niu and Sternberg, 2001; Yi et al., 2013; Tang et al., 2018).

A number of large-scale studies and meta-analyses of the relationship between culture and well-being have been conducted in the last couple of decades. Diener et al.’s (2003) review of studies about personality, culture, and well-being points out “there are multiple pathways to well-being and they are somewhat different across cultures, depending on internalized cultural values (p. 416).” A meta-analysis by Steel et al. (2018) revealed that culture matters for individual and national well-being, but in opposite ways: at the individual level, individualism was negatively correlated with all aspects of well-being, whereas at the national level, it was strongly associated with higher well-being. As the current study focuses on the analysis at the individual level, we also expect a negative relationship between individualism and well-being across the countries, with the largest effect size for the Chinese sample, as contemporary China is still the least individualist country of the three (Taras et al., 2012). Accordingly, we anticipate a moderating effect of individualism on the sequential mediation effect of creativity on well-being:

*H4*: Individualism moderates the mediating effect of creativity on FWB in that the mediating effect is stronger for the participants from less individualistic countries like China.

In terms of SC, Taras et al. (2010) study found a positive relationship between individualism and independence (*ρ* = 0.27, *p* < 0.05, *SD*ρ = 0.17), and between individualism and social avoidance (*ρ* = 0.25, *p* < 0.05, *SD*ρ = 0.15). Therefore, we expect that the mediating effect of creativity between PIC and SC is particularly pronounced in China, but not necessarily in Germany or the United States, as both countries belong to individualistic cultures where independence and autonomy are more emphasized.

*H5*: Individualism moderates the mediating effect of creativity on SC in that the mediating effect is stronger for the participants from less individualistic countries like China.

### The Present Study

**Figure 1:**
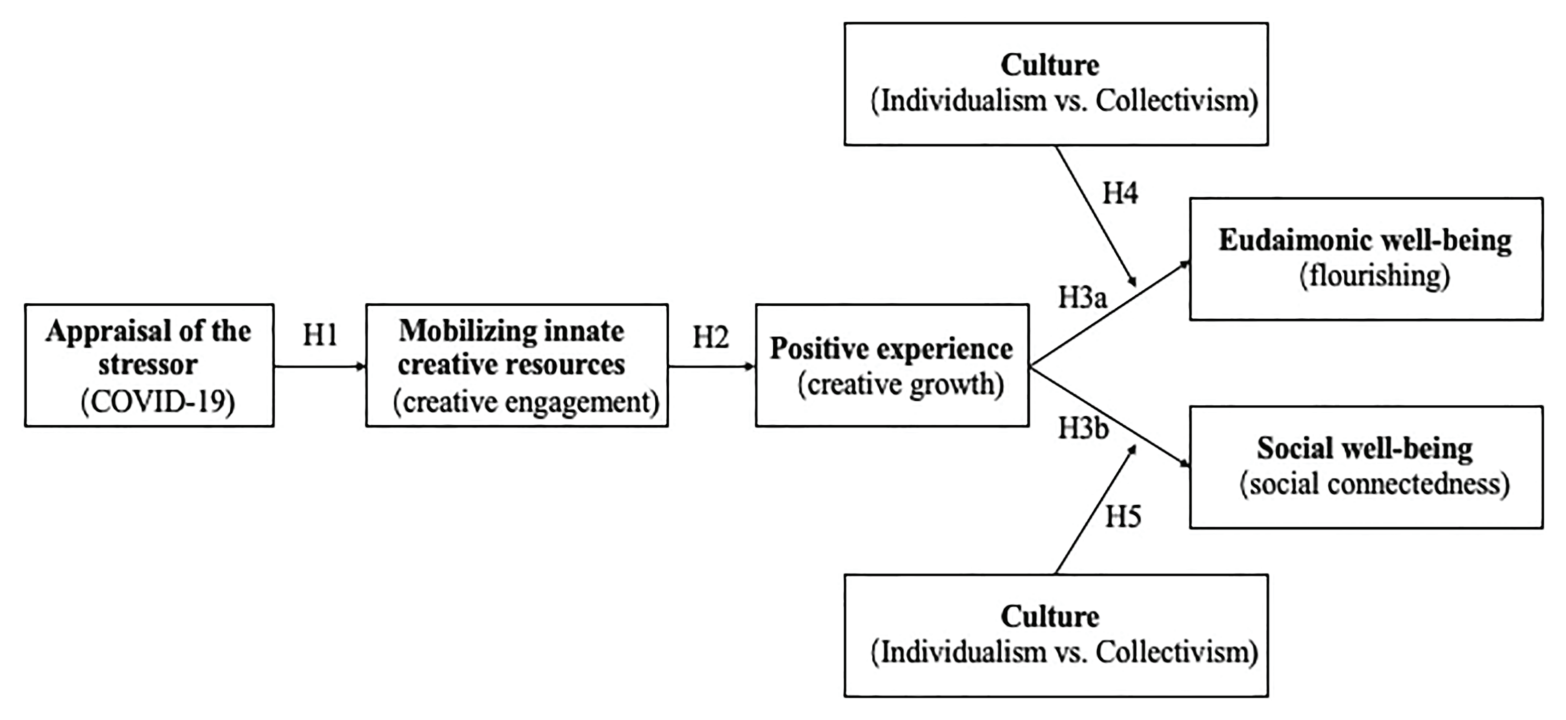
Theoretical model of the study: the culturally moderated mediating model of creativity on well-being and social connectedness (SC).

Recent research has suggested that creativity can be an effective resource for individuals encountering a crisis situation (e.g., Damian and Simonton, 2014; Orkibi and Ram-Vlasov, 2019). In addition, research on the relationship between creativity and well-being as well as SC is sparse but suggests that creativity can be positively related to these outcomes (Smith, 2016; Conner et al., 2018). However, at least two gaps have emerged: first, the few studies that have examined this topic have focused on the influence of either the artistic or everyday creativity on well-being. To our knowledge, no studies have examined the relationship between problem-solving-focused functional creativity (see Cropley and Cropley, 2010) and well-being. Second, the TCM (Corry et al., 2014, 2015) proposes that creativity is a universally applicable transformative coping strategy in stressful situations. However, cross-cultural research on this hypothetically universal mechanism is lacking. The present study attempts to fill these gaps by focusing on functional creativity of employee samples. Following a cross-cultural design, we will examine the culturally moderated mediating model of creativity on well-being and SC (see Figure 1) across three culturally different countries: China, Germany, and the United States.

## Materials and Methods

### Participants

A total of 1,420 part- or full-time employees from China (*n* = 489, 40% females, *M*_age_ = 29.4, *SD* = 5.2), Germany (*n* = 599, 47% females, *M*_age_ = 33.2, *SD* = 11.2), and the United States (*n* = 332, 43% females, *M*_age_ = 38.5, *SD* = 11.6) were recruited for the study. In both China and Germany, most of the participants were from the branches of commercial services, health or social affairs, production and manufacturing, or business organizations; whereas in the United States, most of the participants were from the branches of agriculture or horticulture, media, art, culture, or design. Most of the participants were employed full-time when they participated in the study, including 100% in the German sample, 92.2% in the Chinese sample, and 86.2% in the United States sample.

COVID-19 has hit different regions and countries with different severity, and different regions and countries have taken different measures to prevent the spread of the virus. These differences can cause variations in psychological and behavioral reactions to the crisis. In order to control for the variations that regional differences can cause to the results of the study, we recruited participants from the regions with comparable severity of the pandemic. According to the National Health Commission of China^1^ during May and June 2020 when the data were collected, the provinces of Hubei, Henan, and Guangdong had the highest number of registered COVID-19 cases in China. The data of the Robert Koch Institute^2^ showed that upon data collection, Bavaria, Nordrhein-Westfalen, and Baden Wuerttemberg had the most registered COVID-19 cases in Germany. Participants from the above-mentioned COVID-19 hotpots in China and Germany were recruited for the study. In the United States, participants were not selected by the region because many hotspots existed. By late spring 2020, COVID-19 cases were pervasive across the 50 United States states according to the report of the Centers for Disease Control and Prevention.^3^ Therefore, the “stay-at-home” and “shelter-in-place” mandates were declared for many states during the data collection period.^4^ Although information on participant location (state) was collected, the extent to which a state would be considered a hotpot relative to other states in the months of May and June was unclear.

Table 1 presents a summary of the demographic information of the sample of the study. From this table, we can see that the three samples are fairly comparable in terms of gender and employment status. The Chinese and German samples are also comparable in terms of employment branches and the severity of the pandemic.

**Table 1:** Sample demographic information.

	China (*n* = 489)	Germany (*n* = 599)	United States (*n* = 332)
Gender			
Male	293 (59.9%)	317 (52.9%)	189 (56.9%)
Female	196 (40.1%)	282 (47.1%)	141 (42.5%)
Other	0	0	2 (0.6%)
Age			
Minimum	17	17	20
Maximum	50	66	69
Mean	29.35	33.17	38.51
SD	5.16	11.15	11.57
Employment			
Full-time	451 (92.2%)	599 (100%)	287 (86.2%)
Part-time	38 (7.8%)	0 (0%)	46 (13.8%)
Industry			
Commercial services	141 (28.8%)	112 (18.7%)	20 (6.0%)
Health, social affairs	70 (14.3%)	92 (15.4%)	2 (0.6%)
Production, manufacturing	65 (13.3%)	47 (7.8%)	7 (2.1%)
Business organization	49 (10.0%)	42 (7.0%)	29 (8.7%)
Media, art, culture, design	32 (6.5%)	43 (7.2%)	39 (11.7%)
Agriculture, horticulture	21 (4.3%)	5 (0.8%)	71 (21.4%)
Region	COVID-19 hotspots in China:Hubei: 233 (47.7%) Guangdong: 147 (30.1%) Henan: 109 (22.3%)	COVID-19 hotspots in Germany: Bayern: 511(85.3%) Baden Württemberg: 54 (9.0%) Nordrhein-Westfalen: 34 (5.7)	California: 44 (13.3%) Texas: 25 (7.5%) New York: 24 (7.2%) Florida: 23 (6.9%)

COVID-19, coronavirus disease 2019.

### Measures

#### Perceived Impact of Coronavirus Disease 2019

PIC was measured by two items developed for the purpose of the study, asking about how the participants perceived the impact of COVID-19 on their daily and professional lives. Participants reported on an 11-point Likert scale the degree of the impact with the number ranging from 0 (*no influence at all*) to 10 (*extreme influence*). The internal consistency of the scale is good for the Chinese (*α* = 0.88) and United States samples (*α* = 0.81), whereas somewhat poor for the German sample (*α* = 0.57). The lower internal consistency of this construct in the German sample might be due to the subsidiaries that the German government has allocated to prevent layoffs in certain branches. Such measures may have caused variance in German participants’ perceptions of the impact of COVID-19.

#### Creativity Measures

Two measures of creativity were used in this study. Previous research suggests that measures of creativity may have a significant effect on the relationships identified (Reiter-Palmon and Schoenbeck, 2020). Using two different measures allows for triangulation and compensation. The CPE measures participants’ actual engagement in creative processes and follows the functional creativity approach. The PCG measures perceived increase/growth in creativity or motivation for creativity given a specific potentially traumatic event (such as COVID-19).

CPE in this study is perceived as employees’ involvement in creativity-relevant processes in problem identification, information searching and encoding, and idea and alternative generation. Zhang and Bartol (2010) developed an 11-item CPE scale for their studies about empowering leadership and employee creativity in China. Eight items were selected based on the results of a pretest in China and Germany, in which three items did not perform well in a factor analysis. The eight items include two from the dimensions “problem identification” and “information searching and encoding,” respectively, and four from the dimension “idea generation.” Participants were instructed to rate to what extent they participated in the creative actions during the COVID-19 pandemic, and the items were adapted to the COVID-19 circumstances, such as “I think about the problems caused by Corona virus from multiple perspectives.” The items were rated on a 5-point Likert scale ranging from 1 (*never*) to 5 (*very frequently*). This scale demonstrates good internal consistency for all three samples, with the Cronbach *α* of 0.87 for China and the United States and 0.86 for Germany.

PCG assesses the extent to which participants perceived that their creativity or motivation to engage in creative activities increased as a result of the main event (such as the COVID-19 pandemic; Forgeard, 2013). The six-item PCG scale developed by Forgeard (2013) was used for the present study, with each item being adapted to the COVID-19 situation. One sample item is “The difficult events I experience during the Corona crisis make me a more creative person.” All items were self-rated using a 5-point Likert scale, which ranged from 1 (*not at all*) to 5 (*extremely*). The internal consistency of the measures is high, with the Cronbach *α* of 0.79 for China, 0.87 for Germany, and 0.88 for the United States.

#### Well-Being Measures

The outcome variables of the present study are two types of well-being: eudaimonic well-being (i.e., flourishing) and social well-being (i.e., SC). Flourishinig well-being (FWB), defined as the feelings of meaning, engagement, purpose of life, and optimism (Ryan and Deci, 2001; Diener et al., 2010), was measured by the eight-item Flourishing Scale developed by Diener et al. (2010). This short scale measures important aspects of positive psychological well-being such as self-esteem, purpose, and optimism. Participants were asked to self-rate their status of FWB. One sample item from this scale was “I am engaged and interested in my daily activities.” A 7-point Likert scale, ranging from 1 (*strongly disagree*) to 7 (*strongly agree*), was used for this measure. The internal consistency of the measures is excellent for the Chinese (*α* = 0.90) and United States (*α* = 0.91) samples and good for the German (0.85) sample.

Social connectedness (SC), conceptualized as an individual’s sense of belonging and the subjective perception of having close and distant relationships in the social context (Lee and Robbins, 1998), was measured with the eight-item scale of the SC scale developed by Lee and Robbins (1995). This scale focuses on the emotional distance between self and others in terms of connectedness, affiliation, and companionship. The original items reflected the personal struggle of trying to maintain belongingness with others and were stated in a negative direction such as “I feel disconnected from the world around me.” A reversed 6-point Likert scale with 1 = *strongly agree* to 6 = *strongly disagree* was suggested so that as SC score can be directly produced without reversing the items. However, this *unusual* inversed format of scale was criticized by participants in our pretest because of its inconsistency with all other scales and because it caused confusion. As a result, we reversed the suggested scale description into 1 = *strongly disagree* and 6 = *strongly agree*. In computing the variable, we first summed the values of the items and then reversed the summed score to get the value of SC. The internal consistency of the measures is excellent for all three samples with the Cronbach *α* 0.94, 0.90, and 0.96 for the Chinese, German, and United States samples, respectively.

#### Moderator

*Individualism vs. collectivism* was conceptualized as a *unidimensional* construct, following the tradition of Hofstede’s (1980) original model of culture and the approach widely used in meta-analyses of this cultural dimension (Oyserman et al., 2002; Taras et al., 2010; Steel et al., 2018). This construct was measured with the seven items used by Wang and Liu (2019) in their cross-cultural study involving Australian and Chinese samples. These items focus on whether individuals prioritize the interests of a group over their personal interests. One sample item is “Individuals should sacrifice self-interest for the group.” Participants rated on a 7-point Likert scale ranging from 1 (*strongly disagree*) to 7 (*strongly agree*). For the purposes of consistency with most of the existing literature review or meta-analyses (e.g., Taras et al., 2010; Steel et al., 2018), which focus on individualism, the sum score of this measure was reversed and renamed as “individualism.” The internal consistency of the measures is excellent for the Chinese (Cronbach’s *α* = 0.91) and United States (Cronbach’s *α* = 0.92) samples and good for the German sample (Cronbach’s *α* = 0.83).

### Procedures

One major challenge of cross-cultural study is the equivalence of data, the extent to which the research elements of a study have the same meaning, and can be applied in the same way, in different cultural contexts (Hult et al., 2008). Procedures were taken in the present study, from research design to data collection, to ensure the construct, measurement, and acquisition equivalence of the data from the three participating countries: first, all research instruments except the two-item scale about the impact of the COVID-19 were established and validated scales, and these items were adapted to the COVID-19 situation, which provided the common context for the cross-cultural study. Second, the questionnaire, originally in English, was translated into Chinese and German by applying the team-based collaborative and iterative translation method proposed by Douglas and Craig (2007). The most convenient method of back translation (Brislin, 1980) was not used because of its weakness in assuring the conceptual equivalence and cross-cultural validity of the different versions of the instruments (Douglas and Craig, 2007). Two Chinese-German bilingual translators were involved in the translation by strictly following the steps of the collaborative and iterative translation method. Third, multiple rounds of pretests were conducted to ensure the conceptual equivalence, measurement accuracy, and a smooth conduction of the survey. These pretests also helped to double-check the quality of the translation. In case of “strange” or “difficult” questions of the survey, the translators met again together with the first author of the article, who is trilingual and has a psychological background, to compare the translation with the original items till the best translation was agreed by the three parties. Fourth, to ensure the comparability of the samples, we set a clear sampling frame for the current study and matched the samples from the three countries in terms of gender, age, and employment status. The branch and the severity of the pandemic were also carefully matched for the Chinese and German samples. These procedures were not taken for the United States sample because the spread and impact of the virus were thought to extend all across the United States and because information on the relative severity of the virus among regions was limited.

Data were collected from May to June in the three countries using online survey tools – Wenjuan Xing in China, UniPark in Germany, and Qualtrics (through the MTurk) in the United States. Overall, the participants took about 15 min to complete the questionnaire. To ensure data quality, we applied the following procedures to clean the data: (1) checking for response time such that too fast a response time is indicative of bots or untrustworthy responses. As the pretests show that one needs at least 5 min to complete the survey, participants who spent less than 5 min to answer the questions were excluded from analyses; (2) checking for nonsensical responses to open-ended questions; (3) checking for duplicate IP addresses and coordinate locations; (4) checking for 50% or more incorrect responses to the set of included attention check items (e.g., “Please select ‘Agree’”). If a survey responder failed two or more of these checks, their data were excluded from analyses; and (5) besides, we imbedded three honest response questions suggested by Vésteinsdóttir et al. (2019) to reduce socially desirable responding in the self-reports of the participants.

### Data Analysis Strategies

Data analyses were conducted using SPSS 26 and Mplus 8.4. Before testing the hypotheses, we first employed confirmatory factor analysis (CFA) to establish the measurement model for the key constructs. As the data were collected from three countries, we then conducted a series of multigroup CFAs to evaluate whether the measurement invariance was satisfied. For hypothesis testing, we adopted the path analysis to simultaneously estimate all the effects of the independent variables on our two outcome variables in a single model. We implemented a bootstrapping procedure to estimate and test all indirect effects (Preacher and Hayes, 2008), and we further relied on Edwards and Lambert (2007) strategy to test the moderated mediation effects.

## Results

### Measurement Model and Common Method Variance Test

The hypothesized model consists of six latent variables: PIC, CPE, PCG, individualism (IND), FWB, and SC. Model fit evaluation was conducted using comparative fit index (CFI; acceptable if ≥0.90, and satisfactory when ≥0.95), Tucker-Lewis index (TLI; acceptable if ≥0.90, and satisfactory when ≥0.95), root mean square error of approximation (RMSEA; ≤0.08), and standardized root mean square residual (SRMR; ≤0.08) for an acceptable fit (Schermelleh-Engel et al., 2003). Results of the CFA demonstrated acceptable fit for the hypothesized six-factor model (Table 2). Given that all study variables were measured using self-report Likert-type scales, common method variance (CMV) was examined (Podsakoff et al., 2003, 2012). We took a non-congeneric approach to diagnose the impact of CMV (Richardson et al., 2009). The unmeasured latent method construct technique (Williams et al., 1989, 2010) was used to extract CMV from our six latent constructs. A bi-factor model with a latent factor was specified with loadings from all items constrained to be equal. Fit indices of the bi-factor model did not show a significant improvement in the overall model fit from the six-factor model/baseline model (see Table 2). From these findings, we could conclude that CMV would not bias the analysis significantly. Furthermore, a series of alternative five-factor models were constructed by combining two mediator constructs (i.e., five-factor Model 1 of Table 2), two outcome variables (i.e., five-factor Model 2), and the second mediator with each outcome variable (i.e., five-factor Model 3 and Model 4) to examine whether the measure of each construct could be discriminated from each other. As indicated in Table 2, the hypothesized six-factor model demonstrated the best model fit in comparison to all four alternative models.

**Table 2:** Results of confirmatory factor analysis (CFA) and test for common method variance.

Model	*χ*^2^ (df)	CFI	TLI	RMSEA	SRMR
Six-factor model (hypothesized model)	2,503.176 (614)	0.940	0.935	0.047	0.040
Bi-factor model (with marker variable)	2,494.788 (613)	0.940	0.935	0.046	0.040
Five-factor Model 1 (CPE and CPG combined)	4,317.681 (619)	0.882	0.873	0.065	0.052
Five-factor Model 2 (FWB and SC combined)	6,526.838 (619)	0.812	0.797	0.082	0.119
Five-factor Model 3 (CPG and FWB combined)	6,013.427 (619)	0.828	0.815	0.078	0.093
Five-factor Model 4 (CPG and SC combined)	7,145.698 (619)	0.792	0.776	0.086	0.142

Each of the five-factor models was constructed by combining all items of two constructs into one latent variable. PIC, perceived impact of COVID-19; CPE, creative process engagement; PCG, perceived creative growth; IND, individualism; FWB, flourishing well-being; and SC, social connectedness.

### Measurement Invariance Across Countries

Furthermore, we conducted measurement invariance tests to ensure that the constructs and their relationships are comparable in each country. We used the cutoff criterion of a −0.01 change in CFI and an RMSEA change of 0.015 for evaluating measurement invariance (Cheung and Rensvold, 2002; Chen, 2007). According to the criteria of Putnick and Bornstein (2016), the results show satisfactory configural invariance. In the next step, metric invariance was supported, indicating that the items show similar factor loading patterns across all countries. However, because of scalar non-invariance, our model only demonstrated partial invariance. Results of the measurement invariance test are shown in Table 3.

**Table 3:** Measurement invariance test of the measurement model across the three countries.

Model	*χ*^2^ (df)	CFI	RMSEA	ΔCFI	ΔRMSEA
Six-factor model					
Configural invariance	3,481.56 (1,842)	0.928	0.043	--	--
Metric invariance	3,662.664 (1,904)	0.922	0.044	−0.005	0.001
Scalar invariance	4,662.494 (1,966)	0.881	0.054	−0.041	0.010
Bi-factor model (with marker variable)					
Configural invariance	2,926.066 (1,731)	0.947	0.038	--	--
Metric invariance	3,266.455 (1,865)	0.938	0.04	−0.009	0.002
Scalar invariance	3,815.571 (1,952)	0.917	0.046	−0.021	0.006

ΔCFI and ΔRMSEA of the metric invariance model were calculated against the corresponding configural invariance model. ΔCFI and ΔRMSEA of the scalar invariance model were calculated against the corresponding metric invariance model. CFI, comparative fit index; and RMSEA, root mean square error of approximation.

### Descriptive Statistics

The descriptive statistics, Pearson’s correlation coefficients, and reliability measures for all variables for the pooled sample and by country are presented in Table 4.

**Table 4:** Descriptive statistics, correlations, and reliability of variables.

Variables	*M*	*SD*	1	2	3	4	5	6
Pooled sample (*n* = 1,420)								
1. PIC	7.55	2.06	(0.73)					
2. CPE	3.54	0.73	0.23^***^	(0.89)				
3. PCG	3.62	0.80	0.23^***^	0.57^***^	(0.89)			
4. IND	3.29	1.08	−0.11^***^	−0.35^***^	−0.29^***^	(0.89)		
5. FWB	5.63	0.76	0.06^*^	0.37^***^	0.33^***^	−0.26^***^	(0.89)	
6. SC	4.74	1.21	−0.14^***^	0.04	0.00	0.03	0.40^***^	(0.94)
Chinese sample (*n* = 489)								
1. PIC	7.79	1.81	(0.88)					
2. CPE	3.94	0.54	0.12^**^	(0.87)				
3. PCG	4.04	0.50	0.10^*^	0.68^***^	(0.79)			
4. IND	3.01	1.09	−0.01	−0.47^***^	−0.43^***^	(0.91)		
5. FWB	5.71	0.75	0.12^**^	0.67^***^	0.62^***^	−0.45^***^	(0.90)	
6. SC	4.89	0.94	0.03	0.29^***^	0.29^***^	−0.22^***^	0.52^***^	(0.94)
German sample (*n* = 599)								
1. PIC	7.18	2.07	(0.57)					
2. CPE	3.11	0.67	0.14^**^	(0.86)				
3. PCG	3.23	0.82	0.18^***^	0.31^***^	(0.87)			
4. IND	3.56	0.87	0.02	−0.07	−0.11^**^	(0.83)		
5. FWB	5.55	0.66	0.03	0.22^***^	0.19^***^	−0.08^*^	(0.85)	
6. SC	5.13	0.76	−0.01	0.02	0.07	−0.03	0.51^***^	(0.90)
United States sample (*n* = 332)								
1. PIC	7.87	2.29	(0.81)					
2. CPE	3.71	0.67	0.31^***^	(0.87)				
3. PCG	3.71	0.79	0.29^***^	0.52^***^	(0.88)			
4. IND	3.23	1.26	−0.26^***^	−0.35^***^	−0.22^***^	(0.92)		
5. FWB	5.66	0.93	0.01	0.33^***^	0.32^***^	−0.20^***^	(0.91)	
6. SC	3.80	1.64	−0.27^***^	−0.02	0.00	0.17^***^	0.44^***^	(0.96)

The values in parentheses are the Cronbach *α*. *M*, mean; *SD*, standard deviation; PIC, perceived impact of coronavirus disease 2019 (COVID-19); CPE, creative process engagement; PCG, perceived creative growth; IND, individualism; FWB, flourishing well-being; and SC, social connectedness.

* *p* < 0.05;

** *p* < 0.01;

*** *p* < 0.001.

PIC was positively correlated with CPE with the strongest correlation in the United States sample, *r* = 0.31, *p* < 0.001. Thus, H1 is supported. CPE and PCG were significantly correlated with each other with *r* ranging from 0.31 to 0.68, *p* < 0.001, providing support to H2. Both creative measures were also positively correlated with FWB to a moderate degree with *r* ranging from 0.19 to 0.67, *p* < 0.001, thus providing support for H3a. These results also indicate that the possible mediating effect of CPE and creative growth between the COVID-19 impact and FWB can be assumed. All the above-mentioned results were significant across three countries. In contrast to these consistent results, correlations to SC revealed a somewhat different picture: the two creativity measures were only positively correlated with SC in the Chinese (for both, *r* = 0.29, *p* < 0.001) sample, but not the German or United States samples. Thus, H3b was supported only in the Chinese sample.

IND was negatively correlated with FWB in all three countries, with the strongest correlation in the Chinese sample, *r* = −0.45, *p* < 0.001. IND was also negatively correlated with SC, but this relationship was only significant in the Chinese (*r* = −0.22, *p* < 0.001) and United States samples (*r* = 0.17, *p* < 0.001), but not the German sample. These results indicate that there might be nuanced differences in the mediation models for FWB and SC.

### Serial Mediation Analysis With the Pooled Data

**Figure 2:**
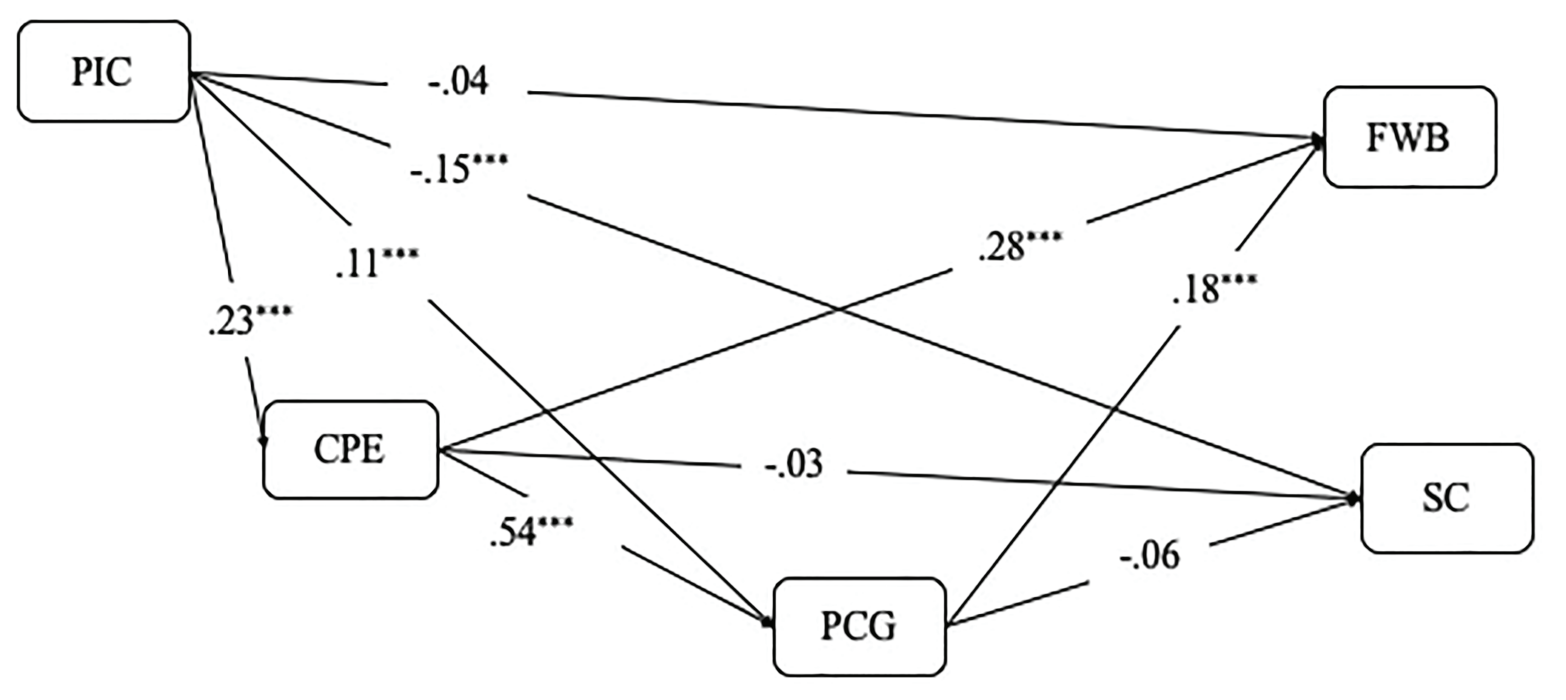
Path estimates for the pooled sample of perceived impact of coronavirus disease 2019 (PIC COVID-19) on flourishing well-being (FWB) and SC mediated by creative process engagement (CPE) and perceived creative growth (PCG). ^***^p < 0.001.

To further examine the direct and indirect effects of creativity in our models, we implemented a bootstrapping procedure with 10,000 bootstraps following Preacher and Hayes (2008). Indirect effects were considered as significant if the 95% CI of the indirect effect estimate did not contain zero. In the first step, we conducted the mediation analysis for the pooled sample before examining model differences between samples. The overall serial mediation model is presented in Figure 2.

Results of the indirect effects of the serial mediation model are shown in Table 5. When FWB was the focal outcome variable, both the total indirect effect of COVID-19 impact on FWB and each of the specific indirect effects were significant. The hypothesized serial mediation effect for COVID-19 impact on FWB through CPE and creative growth was also significant (indirect effect = 0.008, 95% CI = 0.005, 0.013). However, neither the total indirect effect of COVID-19 impact on SC nor any specific indirect effect related to it was significant, including the serial mediation effect through CPE and creative growth on SC (indirect effect = 0.004, 95% CI = 0.000, 0.009). Thus, the results indicate that CPE and creative growth only mediate the relationship between COVID-19 impact and FWB in the pooled sample.

**Table 5:** Indirect effects of PCI on FWB and SC for the pooled sample.

Paths	Estimate	SE	95% CI	
			Lower	Upper
Outcome variable: FWB				
PIC→CPE→FWB	0.024	0.004	0.016	0.033
PIC→PCG→FWB	0.007	0.002	0.004	0.013
PIC→CPE→PCG→FWB	0.008	0.002	0.005	0.013
Total indirect effect of PIC on FWB	0.040	0.006	0.030	0.051
Outcome variable: SC				
PIC→CPE→SC	−0.004	0.004	−0.013	0.004
PIC→PCG→SC	0.004	0.002	0.000	0.009
PIC→CPE→PCG→SC	0.004	0.002	0.000	0.009
Total indirect effect of PIC on SC	0.004	0.004	−0.005	0.013

PIC, perceived impact of coronavirus disease 2019 (COVID-19); CPE, creative process engagement; PCG, perceived creative growth; FWB, flourishing well-being; SC, social connectedness; Estimate, standardized path coefficients; and *SE*, standard error.

### Multigroup Serial Mediation Analysis

**Figure 3:**
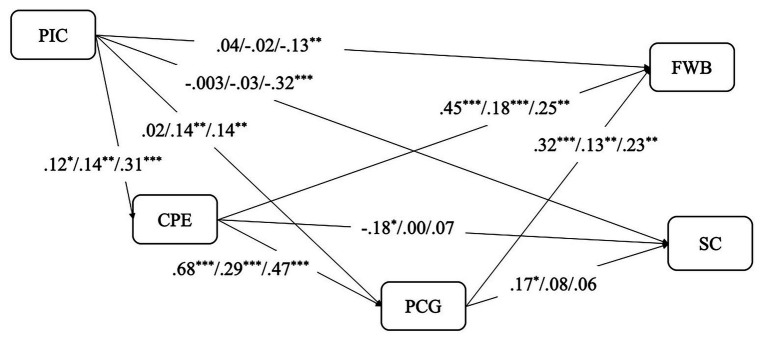
Path estimates for the Chinese, German, and United States samples of PIC on FWB and SC mediated by CPE and PCG. ^*^p < 0.05; ^**^p < 0.01; ^***^p < 0.001. Standardized path coefficients. For each path, the first, second, and third coefficients are the estimate of Chinese, German, or United States sample, respectively.

In order to examine group differences in the indirect effects of the creativity variables, we conducted multigroup serial mediation analyses with country as the grouping variable (China vs. Germany vs. the United States). Path estimates are shown in Figure 3. As indicated in Figure 3, while the effects of CPE and PCG on FWB are significant in all three samples, the effects of CPE and PCG on SC are only significant in the Chinese sample but not in the German or United States sample.

All indirect effects of the multigroup serial mediation model are shown in Table 6. When FWB was the focal outcome, the total indirect effect of COVID-19 impact on FWB for all three samples was positive and significant, though the PIC→CPE→PCG→FWB path was significant only for the Chinese (indirect effect = 0.010, 95% CI = 0.002, 0.021) and United States samples (indirect effect = 0.014, 95% CI = 0.004, 0.030), but not for the German sample. However, when the focal outcome was SC, both the total indirect effect of COVID-19 impact on FWB (indirect effect = 0.019, 95% CI = 0.003, 0.040) and the PIC→CPE→PCG→SC path (indirect effect = 0.007, 95% CI = 0.001, 0.020) were significant for the Chinese sample. For the United States sample, although the total indirect effect (indirect effect = 0.019, 95% CI = 0.003, 0.040) was significant, none of the three specific indirect effects were significant. Neither the total indirect effect of COVID-19 impact on FWB nor any effect of the specific indirect paths was significant for the German sample. To summarize, there are abundant variations in terms of path coefficients and indirect effects across three countries. It is also worth noting that such cultural variations seemed to be more salient concerning the effects on SC compared with FWB.

**Table 6:** Indirect effects of PCI on FWB and SC for China, Germany, and the United States.

Paths	Estimate	SE	95% CI	
			Lower	Upper
Chinese sample				
Outcome variable: FWB				
PIC→CPE→FWB	0.022	0.010	0.003	0.042
PIC→PCG→FWB	0.002	0.005	−0.008	0.013
PIC→CPE→PCG→FWB	0.010	0.005	0.002	0.021
Total indirect effect of PIC on FWB	0.035	0.015	0.004	0.062
Outcome variable: SC				
PIC→CPE→SC	0.011	0.006	0.002	0.026
PIC→PCG→SC	0.001	0.004	−0.005	0.011
PIC→CPE→PCG→SC	0.007	0.005	0.001	0.020
Total indirect effect of PIC on SC	0.019	0.009	0.003	0.040
German sample				
Outcome variable: FWB				
PIC→CPE→FWB	0.008	0.003	0.003	0.016
PIC→PCG→FWB	0.006	0.003	0.002	0.014
PIC→CPE→PCG→FWB	0.002	0.001	0.000	0.004
Total indirect effect of PIC on FWB	0.016	0.005	0.008	0.027
Outcome variable: SC				
PIC→CPE→SC	0.000	0.003	−0.005	0.005
PIC→PCG→SC	0.004	0.003	0.000	0.013
PIC→CPE→PCG→SC	0.001	0.001	0.000	0.004
Total indirect effect of PIC on SC	0.005	0.004	−0.001	0.014
United States sample				
Outcome variable: FWB				
PIC→CPE→FWB	0.032	0.012	0.013	0.061
PIC→PCG→FWB	0.013	0.007	0.003	0.033
PIC→CPE→PCG→FWB	0.014	0.007	0.004	0.030
Total indirect effect of PIC on FWB	0.059	0.014	0.033	0.089
Outcome variable: SC				
PIC→CPE→SC	0.017	0.015	−0.011	0.048
PIC→PCG→SC	0.007	0.007	−0.003	0.026
PIC→CPE→PCG→SC	0.007	0.007	−0.005	0.023
Total indirect effect of PIC on SC	0.030	0.017	0.003	0.070

PIC, perceived impact of coronavirus disease 2019 (COVID-19); CPE, creative process engagement; PCG, perceived creative growth; FWB, flourishing well-being; SC, social connectedness; Estimate, standardized path coefficients; and *SE*, standard error.

We conducted a series of multigroup analyses to further explore the group differences in the indirect associations between the variables. Specifically, the Chinese sample served as the reference group; a given path was constrained to be equal across the reference group (i.e., the Chinese sample) and the other group (i.e., the German or United States sample). The Δ*χ*^2^ of the constrained model was calculated and tested against the un-constrained, freely estimated model. A significant Δ*χ*^2^ indicates that the path coefficients for the two groups are different and should not be constrained equal. Results of the serial model comparisons are presented in Table 7. As can be seen, Δ*χ*^2^ was significant for each of the four paths between the Chinese and German samples. For the Chinese and United States samples, while the constrains of two paths related to SC resulted in non-significant *χ*^2^ changes, those related to FWB led to significant *χ*^2^ changes. Thus, the results indicate that the proposed serial indirect effects exhibited substantial cultural variations between Eastern (e.g., China) and Western countries (e.g., Germany and the United States).

**Table 7:** Model comparison results of multigroup analyses.

Path constrained to be equal	Δ*χ*^2^	Δdf	*p*
Chinese vs. German sample			
CPE→FWB	36.243^***^	1.000	0.000
PCG→FWB	23.395^***^	1.000	0.000
CPE→SC	7.433^**^	1.000	0.006
PCG→SC	4.204^*^	1.000	0.040
Chinese vs. United States sample			
CPE→FWB	7.167^**^	1.000	0.007
PCG→FWB	4.211^*^	1.000	0.040
CPE→SC	0.498	1.000	0.480
PCG→SC	1.149	1.000	0.284

Chinese sample was set as the reference group. For each constrained model, the Δ*χ*^2^ was calculated and tested against its un-constrained model. CPE, creative process engagement; PCG, perceived creative growth; FWB, flourishing well-being; and SC, social connectedness.

* *p* < 0.05;

** *p* < 0.01;

*** *p* < 0.001.

### Moderated Serial Mediation Model With Individualism as the Moderator

Given that there were cultural variations in CPE→FWB/SC and PCG→FWB/SC paths (see Table 7), IND was evaluated as a moderator of the paths from both CPE and PCG to outcome variables (i.e., FWB and SC). We employed Edwards and Lambert (2007) strategy to estimate the indirect effects at one *SD* above and below the mean of IND and to further test the differences for the indirect effects based on CIs derived from bootstrap estimates. As indicated in Figure 1, the conditional serial mediation effects were of interest to our current study. Serial moderated mediation results are presented in Table 8. It can be seen that the serial mediation effect of creativity on FWB was significant when IND was low (indirect effect = 0.013, 95% CI = 0.008, 0.019) rather than high (indirect effect = 0.004, 95% CI = −0.001, 0.010). Furthermore, the difference of such indirect effect between low vs. high value of IND was also significant (index of moderated mediation = −0.004, 95% CI = −0.008, −0.001). Similarly, the serial mediation effect of PIC on SC was significant when IND was low (indirect effect = 0.012, 95% CI = 0.006, 0.021) rather than high (indirect effect = 0.000, 95% CI = −0.006, 0.006). Also, the conditional indirect effects at low vs. high value of IND were significant (index of moderated mediation = −0.006, 95% CI = −0.011, −0.002). Taken together, the above results provided support for H5 and H6.

**Table 8:** Serial moderated mediation results in the pooled sample.

Path	Values of individualism	Estimate	*SE*	95% CI	
				Lower	Upper
PIC→CPE→PCG→FWB	Low (−1 *SD*)	0.013	0.003	0.008	0.019
	Medium (mean)	0.008	0.002	0.005	0.013
	High (+1 *SD*)	0.004	0.003	−0.001	0.010
	IMM	−0.004	0.002	−0.008	−0.001
PIC→CPE→PCG→SC	Low (−1 *SD*)	0.012	0.004	0.006	0.021
	Medium (mean)	0.006	0.002	0.002	0.012
	High (+1 *SD*)	0.000	0.003	−0.006	0.006
	IMM	−0.006	0.002	−0.011	−0.002

PIC, perceived impact of coronavirus disease 2019 (COVID-19); CPE, creative process engagement; PCG, perceived creative growth; FWB, flourishing well-being; SC, social connectedness; Estimate, standardized path coefficients; *SE*, standard error; and IMM, index of moderated mediation.

## Discussion

The present study was driven by two major questions: is creativity a means to well-being and social connectedness when facing crisis such as COVID-19 pandemic? And is this mediating effect of creativity between crisis and well-being/SC universal across different countries? Using comparable employee samples from China, Germany, and the United States, our study reveals a consistent pattern: in all three countries, the perceived impact of COVID-19 triggers creative process engagement, which strengthens employees’ self-reported creative growth, and this leads to a higher level of perceived well-being. As human beings, everybody has the need to reach optimal functioning as such flourishing – the feelings of meaning, engagement, purpose of life, and optimism (Ryan and Deci, 2001; Diener et al., 2010). This need is even more pressing in the face of crisis, as the occurrence of crisis threatens individual’s subjective sense of self and its existential core (Pauchant and Mitroff, 1992). Existing studies have shown, on the one hand, that diversifying or adverse experiences such as mental illness, social rejection/isolation, childhood traumatic experiences, or setbacks in adulthood are associated with a higher level of creativity (for a review, see Damian and Simonton, 2014). On the other hand, creativity has been shown to have a healing effect for victims coping with natural or man-made disasters, and it facilitates posttraumatic growth (e.g., Orkibi and Ram-Vlasov, 2019). Our study supports the positive coping effect of creativity in stressful situations proposed by the TCM (Corry et al., 2014, 2015). Further, our study extends the healing/coping effect of creativity in times of a worldwide pandemic, which influences a broad population across countries. Our study suggests that for people, creativity is an effective way to deal with crisis and to achieve flourishing experiences, and this mediating effect is significant for three historically and culturally different countries. In the present study, we focused on a broad spectrum of creative process engagement and the perceived creative growth and found a sequential mediation from creative process engagement to creative growth. This is a significant extension to a recent study (Conner et al., 2018), which found that everyday creative expressions promote people’s flourishing, and this positive effect is even sustained for the next day. Our study shows that both actual creative engagement and perceived creative growth contribute to the experience of flourishing.

Our study also indicates that the positive mediating effect of creativity is more pronounced for less individualistic people. In particular, the effect through creative process engagement and creative growth on flourishing well-being or social connectedness was the strongest for people with low levels of individualism and non-significant for people with high levels of individualism. This result is consistent with Steel et al.’s (2018) recent meta-analysis about the relationship between culture, wealth, and well-being. In this study, the authors compared the relationship between individualism and well-being using both a large-scale individual-level data set (*n* = 8,438) and a nation-level data set composed of 44 meta-analytic effects representing 1,230 original data points. They found that the relationship between individualism and well-being is negative at the individual level whereas positive at the national level. This result can be explained by the belongingness hypothesis (Baumeister and Leary, 1995) and the social capital theory (e.g., Helliwell et al., 2014; Lange, 2015). The belongingness hypothesis postulates that being an accepted member of a group is a fundamental need of human being. Social capital theory argues that social capital (i.e., supporting social resources, network, relations, and trust) is of vital importance for happiness and job satisfaction particularly in times of crisis. Furthermore, the moderation effect of individualism might be explained by the nature of the stressor (COVID-19) in our model. COVID-19 is a highly social problem, with solutions particularly dependent on how social communities help and support each other (i.e., work together as a collective). Further, one important solution to the spread is that of social distancing, a situation that has an important effect on feelings of SC. Thus, the more collectivistic people behave in this situation, the better they protect their community from getting infected. In a study based on social media data in China, collectivism was found to predict people’s intention to protect themselves and their surroundings from COVID-19 (Huang et al., 2020). In a recent study during COVID-19 in India, Ahuja et al. (2020) demonstrated that collectivism is positively associated with well-being.

In addition, our study shows that creativity also mediates the relationship between perceived impact of the pandemic and the perceived SC, but this relationship was only found for the Chinese sample. Engaging in creative activities in times of crisis (e.g., COVID-19) can help people, particularly those in a less individualistic culture, to cope with difficult events through increased feeling of being socially connected. Glăveanu (2020) pointed out that when investigating creativity in a cultural context, the role and function of communities should be taken into consideration. People of more collectivistic cultures see group membership as a central aspect of their personal identity, value personal traits that reflect collective goals, and derive life satisfaction from successfully carrying out social roles (for a review, see Oyserman et al., 2002). As a result, they might be more active using creativity to strengthen their “social capital” and foster their social relationships. Our study reconfirms the results of the previous study and indicates that, in times of crisis, values associated with individualism and autonomy do not appear to be beneficial in terms of well-being or SC at the individual level no matter if the person is from a more individualistic or collectivistic culture.

Taken together, the current study shows that engaging in creative activities in times of crisis (such as the COVID-19 pandemic) can be more helpful for individuals in collectivistic country (like China) to obtain personal flourishing than for their counterparts in a more individualistic countries (like Germany and the United States). For the former, relying on creativity to cope with difficult events during the pandemic is in parallel to promote well-being and also helps them gain an increased feeling of being socially connected. These findings provide support to the theories about the relationship between culture and well-being. For example, the review of Diener et al. (2003) stresses that there are both universal and culture-specific causes of well-being. In a similar vein, De Dreu (2010) maintains that “Cultural background shapes what is important to the individual, what should be considered relevant issues and problems, what constitutes threats and opportunities (p. 443).”

### Limitations and Future Studies

The findings of the present study should be interpreted and applied with caution, given the limitations of the study. First, the study relied exclusively on the self-report measures. Self-report measures are prone to methodological restrictions such as the influence of personal biases, motivations, differences in understanding of questions, and differences in response styles (Smith, 2004; Harzing, 2006). Future studies should consider adopting a multi-source and repeated measure research design (Taras et al., 2010) with the aim to more effectively eliminate assessment variance that resulted from temporary person- or group-level influences.

Second, the cross-sectional design of the study poses an additional challenge to the threat of the common method bias (Podsakoff et al., 2003). Though we have taken appropriate measures to ensure the common method invariance of the measures, this design did not allow a comprehensive examination of the proposed moderated mediation model and inference of causality. Further experimental or longitudinal studies are called to further examine the validity of the model.

Third, the three samples were quite comparable in terms of age, gender, and employment status, and the Chinese and German samples were also rather comparable in terms of branches, however, the United States sample was not of optimal comparability with the other two samples in terms of the proportion of participating branches. Therefore, results related to the comparison between the United States and the other two countries should be interpreted with caution. Despite this, the rigorous data cleaning and statistical procedures taken for ensuring the measurement invariance across the three countries should have compensated more or less for this limitation.

In addition, though individualism-collectivism has been the most popular cultural dimension in cross-culture studies, other cultural dimensions may be relevant to understanding the relationship between perceived crisis, creativity, and well-being (e.g., Taras et al., 2010). Future cross-cultural studies should include more cultural measures, not necessarily limited to values (cf. Leung and Bond, 2004; Taras and Steel, 2009). For example, the synthesized approach proposed by Beugelsdijk and Welzel (2018) dimensional concept of culture of Hofstede (1980) and dynamic theory of culture of Inglehart (1990, 1997) and Inglehart and Welzel (2005) provides a promising method to understand phenomena across cultures.

## Conclusion

The COVID-19 pandemic has resulted in widespread mental health problems such as anxiety, depression, and posttraumatic stress disorder (Fitzpatrick et al., 2020; Xin et al., 2020). Therefore, among several other priorities, “here is an urgent need for research to address how mental health consequences for vulnerable groups can be mitigated under pandemic conditions” (Holmes et al., 2020, p. 547). The present study reacts to the need of the current challenge the whole world is facing and examines the possible mediating effect of creativity between crisis and well-being. Though this relationship appears to be ubiquitous, this crisis-creativity-well-being relationship has not been sufficiently examined across countries. The current study is among the first to empirically test this relationship cross-culturally. With data from China, Germany, and the United States, we could empirically prove the healing/coping effect of creativity in face of crisis across countries. That is, no matter whether they are from more individualistic or collectivistic cultures, people benefit from the engagement in creativity in helping them achieve positive, flourishing experiences. This mediating effect is even stronger for those from less individualistic countries. Moreover, people from more collectivistic countries, in addition to their flourishing experiences, also feel more socially connected through the help of creative engagement and creative growth. The results of the study add evidence to the positive hidden potential of creativity (Richards, 2007; Kaufman, 2018), thus having profound implications for crisis management, personal development, and positive functioning of individuals and society.

## Data Availability Statement

The raw data supporting the conclusions of this article will be made available by the authors, without undue reservation.

## Ethics Statement

Ethical review and approval was not required for the study on human participants in accordance with the local legislation and institutional requirements. The patients/participants provided their written informed consent to participate in this study.

## Author Contributions

MT and SH designed and directed the project, conceived the original idea, performed the analytic calculations, and wrote the manuscript with support from RR-P, XB, and VM. RR-P, XB, and VM contributed to the design and implementation of the research, to the analysis of the data, and to the writing of the manuscript. All authors contributed to the article and approved the submitted version.

### Conflict of Interest

The authors declare that the research was conducted in the absence of any commercial or financial relationships that could be construed as a potential conflict of interest.

## Footnotes

^1^https://news.qq.com/zt2020/page/feiyan.htm#/?pool=hb&nojump=1

^2^https://www.rki.de/DE/Content/InfAZ/N/Neuartiges_Coronavirus/nCoV.html

^3^https://www.cdc.gov/mmwr/volumes/69/wr/mm6915e4.htm?s_cid=mm6915e4_w

^4^https://www.finra.org/rules-guidance/key-topics/covid-19/shelter-in-place
